# Conserved developmental trajectories of the cecal microbiota of broiler chickens in a field study

**DOI:** 10.1093/femsec/fiac090

**Published:** 2022-07-25

**Authors:** Jannigje G Kers, Francisca C Velkers, Egil A J Fischer, J Arjan Stegeman, Hauke Smidt, Gerben D A Hermes

**Affiliations:** Department Population Health Sciences, Faculty of Veterinary Medicine, Division Farm Animal Health, Utrecht University, Yalelaan 7, 3584 CL Utrecht, The Netherlands; Laboratory of Microbiology, Wageningen University & Research, Stippeneng 4, 6708WE Wageningen, The Netherlands; Department Population Health Sciences, Institute for Risk Assessment Sciences (IRAS), Utrecht University, Yalelaan 2, 3584 CM Utrecht, The Netherlands; Department Population Health Sciences, Faculty of Veterinary Medicine, Division Farm Animal Health, Utrecht University, Yalelaan 7, 3584 CL Utrecht, The Netherlands; Department Population Health Sciences, Faculty of Veterinary Medicine, Division Farm Animal Health, Utrecht University, Yalelaan 7, 3584 CL Utrecht, The Netherlands; Department Population Health Sciences, Faculty of Veterinary Medicine, Division Farm Animal Health, Utrecht University, Yalelaan 7, 3584 CL Utrecht, The Netherlands; Laboratory of Microbiology, Wageningen University & Research, Stippeneng 4, 6708WE Wageningen, The Netherlands; Laboratory of Microbiology, Wageningen University & Research, Stippeneng 4, 6708WE Wageningen, The Netherlands

**Keywords:** 16S rRNA, gut, intestinal, microbial community, microbiome, poultry

## Abstract

There is great interest in identifying gut microbiota development patterns and underlying assembly rules that can inform strategies to improve broiler health and performance. Microbiota stratification using community types helps to simplify complex and dynamic ecosystem principles of the intestinal microbiota. This study aimed to identify community types to increase insight in intestinal microbiota variation between broilers and to identify factors that explain this variation. A total of 10 well-performing poultry flocks on four farms were followed. From each flock, the cecal content of nine broilers was collected at 7, 14, and 35 days posthatch. A total of two robust community types were observed using different clustering methods, one of which was dominated by 7-day-old broilers, and one by 35-day-old broilers. Broilers, 14-day-old, were divided across both community types. This is the first study that showed conserved cecal microbiota development trajectories in commercial broiler flocks. In addition to the temporal development with age, the cecal microbiota variation between broilers was explained by the flock, body weight, and the different feed components. Our data support a conserved development of cecal microbiota, despite strong influence of environmental factors. Further investigation of mechanisms underlying microbiota development and function is required to facilitate intestinal health promoting management, diagnostics, and nutritional interventions.

## Teaser

Conserved cecal microbiota development trajectories across different commercial broiler flocks despite the known strong influence of environmental factors.

## Introduction

The intestinal microbiota is associated with the health and production performance of broiler chickens (Stanley et al.2014a, 2016, Han et al. 2016, Johnson et al. 2018). Therefore, there is great interest in identifying the biological principles that underlie the structure and function of these microbiological ecosystems. This knowledge can contribute to the development of beneficial nutritional management as well as diagnostic tools, to improve broiler health. However, several studies in broilers have described the intestinal microbiota as highly variable within and between repeated experiments (Stanley et al. 2013, Thibodeau et al. 2015, Cuperus et al. 2018). Feed, antimicrobial products, host, and environmental factors have been shown to attribute to the variation in intestinal microbiota (Apajalahti et al. 2001, Borda-Molina et al. 2018, Kers et al. 2019). The effect of factors can overlap as we showed that the effect of a feed intervention on the composition of broiler microbiota was highly dependent on the environment (Kers et al. 2019). In humans, it has also been shown that similar foods can have different effects on the microbiota (Johnson et al. 2019). Therefore, it is important to identify which factors influence the microbiota composition of broilers, and to which extent.

It has been proposed that microbial communities coevolve with their hosts (Dethlefsen et al. 2008, De Filippo et al. 2010, Uhr et al. 2019). Although it is generally accepted that the composition of the intestinal microbiota is unique per individual, conserved compositional patterns, termed enterotypes, were discovered across human adults, independent of age, gender, cultural background, and geography (Arumugam et al. 2011). This enterotype concept was further refined, acknowledging that statistical support for robust clusters was variable and a range of confounding factors could affect the initially defined discrete clusters (Costea et al. 2018). Nevertheless, stratification using cluster analysis can still serve as a powerful tool to reduce the complexity of the microbiota community landscape (Costea et al. 2018). In human infants and adults, community types have been associated with differences in microbial functionality, diseases, and with differences in diet (Arumugam et al. 2011, Costea et al. 2018, Borewicz et al. 2019, Zhong et al. 2019). There have also been attempts to define microbiota community types in poultry. In 31 broilers aged 56 days, the fecal microbiota was classified into four potential community types based on principal component analysis (Kaakoush et al. 2014). These community types or clusters were defined as dominated by *Firmicutes* alone, or in combination with *Proteobacteria*, *Actinobacteria*, or *Bacteroidetes*, respectively (Kaakoush et al. 2014). Another study observed three community types in duodenal content of broilers aged 77 days. One cluster was dominated by *Bacteroides* and *Escherichia–Shigella*, one by *Ochrobactrum* and *Rhodococcus*, and the third by *Bacillus* and *Akkermansia* (Yuan et al. 2020). In addition to community types to observe certain developmental patterns, maturation patterns have been described before. An experimental study showed the maturation of the fecal microbiota until day 30 (Gao et al., 2017), however, if this process contains different phases and is influenced by factors other than prebiotics or antibiotics is unknown.

Although the cecal microbiota has been widely investigated because of its functionality, which in broilers is especially related to the fermentation of feed (Stanley et al. 2014b, Svihus 2014), the factors that contribute to the normal compositional variation in healthy broiler populations have remained under-investigated. Therefore, the main drivers of cecal microbiota variation remain unknown. In humans, factors such as stool consistency and medication have been shown to be important determinants of adult fecal microbiota variation (Falony et al. 2016, Müller et al. 2020). However, broilers have a short life span, which increases the difficulty to identify the factors that influence their microbiota and disentangle these from temporal variation, because the microbiota is most likely still developing at the end of their lives because broilers do not reach adulthood. In human the principles of community types have been studied before. Infants aged 3 to 46 months, ten community types were observed and described in a transition model consisting of three phases: a developmental, transitional and stable phase (Stewart et al. 2018). Another study showed that infants belonging to different microbial clusters also had different degradation patterns of human milk oligosaccharides (Borewicz et al. 2019). Although there is a large difference between the birth of a child and hatching of commercial chickens in terms of exposure, the concept of microbial clusters can provide useful insights in the intestinal microbiota development, as well as into factors that can affect this development and their respective importance.

To this end, this study aimed to explore whether stratification of the cecal microbiota into community types could provide insight into the developmental patterns of cecal microbiota. In addition, we aimed to identify variables impacting this development within and between broiler flocks. A longitudinal study in four well-performing broiler farms with a total of 10 flocks was performed. From each flock, nine individual broilers were sampled at an age of 7, 14, and 35 days posthatch. In total, the cecal microbiota of 270 broilers was determined by 16S ribosomal RNA (rRNA) gene amplicon sequencing. The outcomes of community type analyses have been shown to be highly dependent on the applied clustering algorithms (Koren et al. 2013, Costea et al. 2018). To robustly define community types within the cecal microbiota, two clustering methods were used; partitioning around medoid (PAM) with four beta diversity metrics, and Dirichlet Multinomial Mixtures (DMM). In addition to the community types, host characteristics, environmental factors, and feed components were included in multivariate distance-based redundancy analysis (db-RDA), to study the relative impact of these factors on the variation in microbiota composition between broilers.

## Results

### Microbiota stratification into community types

**Figure 1.**
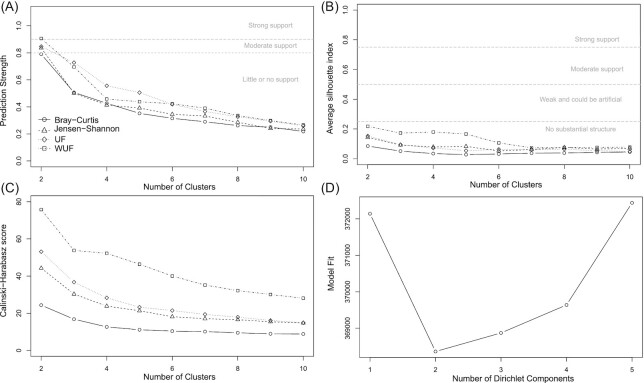
Quality and confidence scores for PAM and DMM clustering of cecal broiler microbiota. The dataset contained 270 broilers of age 7, 14, and 35 days old. All four figures show most support for two clusters. The thresholds for significance of clustering scores are indicated as dashed lines on the plots. **(A)** Based on the prediction strength, strong support was observed for the PAM weighted UniFrac (PAM–WUF), and moderate support when using other distance metrics (BC, JS, and UF). **(B)** No support for two clusters was observed based on the silhouette index, although the highest score was also observed for two clusters. **(C)** All distance metrics showed the highest score for two clusters based on the Calinski–Harabasz score. The PAM–WUF methods showed again the highest score for two clusters. **(D)** The DMM cluster score showed also highest evidence for two clusters.

**Figure 2.**
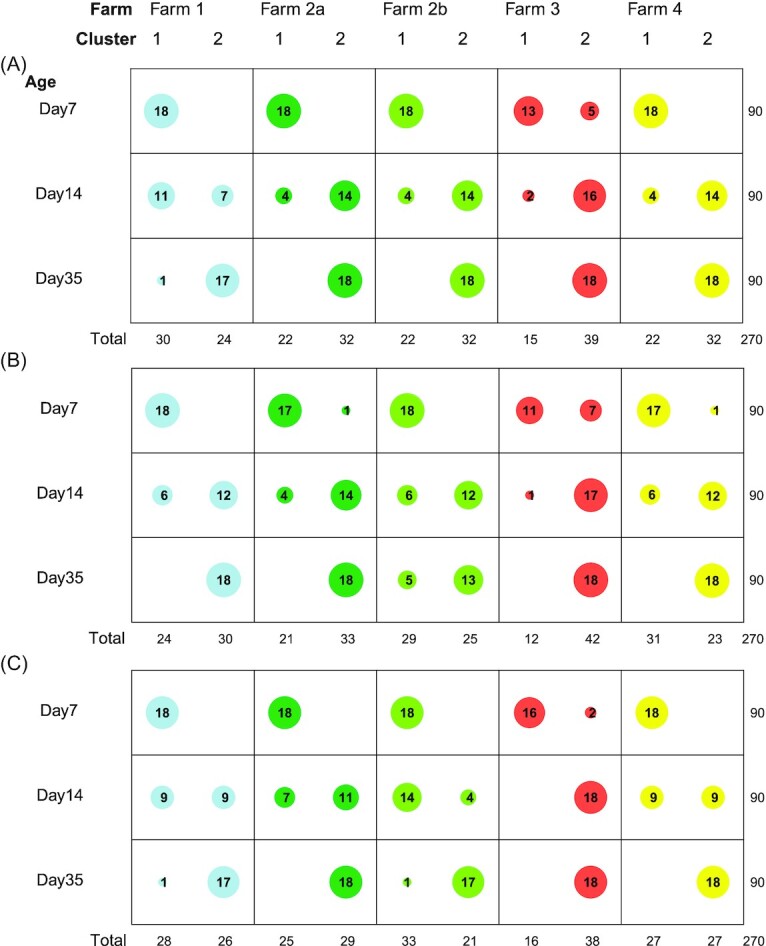
Distribution of clusters across farms and animal age. **(A)** Clusters were assigned using PAM–UF. **(B)** Clusters were assigned using PAM–WUF clustering. **(C)** Clusters were assigned using DMM clustering. The variation of individual samples through different cluster methods stratified per farm. Farm 1 is light blue, Farm 2 is dark green and light green (a and b are different production cycles), Farm 3 is red, and Farm 4 is yellow.

Identifying community types in compositional datasets not only depends on the data itself, but can also be sensitive to the applied methods, therefore, different methods are applied; PAM-based methods using amplicon sequence variants (ASVs) Jensen–Shannon divergence (PAM–JSD), Bray–Curtis dissimilarity (PAM–BC), unweighted UniFrac (PAM–UF), or weighted UniFrac (PAM–WUF), and DMM clustering. Nonetheless, all five applied clustering methods were in concordance, indicating an optimum of two clusters within the 270 broiler chickens aged 7, 14, and 35 days (Fig. 1). Across all five methods, the two clusters were associated with age. Cluster 1 contained almost all 7-day-old broilers, and cluster 2 contained all 35-day-old broilers, whereas the 14-day-old broilers were distributed across both clusters. However, depending on the applied method individual broilers were classified in different clusters (Fig. 2). For example, on day 14 of Farm 1, PAM–UF clustering resulted in 11/18 broilers in cluster 1, PAM–WUF clustering resulted in 6/18 broilers in cluster 1, and DMM clustering resulted in 9/18 broilers in cluster 1 (Fig. 2).

The predominant families were *Lachnospiraceae* in cluster 1 and *Ruminococcaceae* in cluster 2. The predominant genera were *Ruminococcus torques* group in cluster 1 and *Faecalibacterium* in cluster 2 (Figure S1, Supporting Information). The top 10 ASVs, that significantly differed in relative abundance between the two clusters, were independent of the applied clustering method (Table S1, Supporting Information).

**Figure 3.**
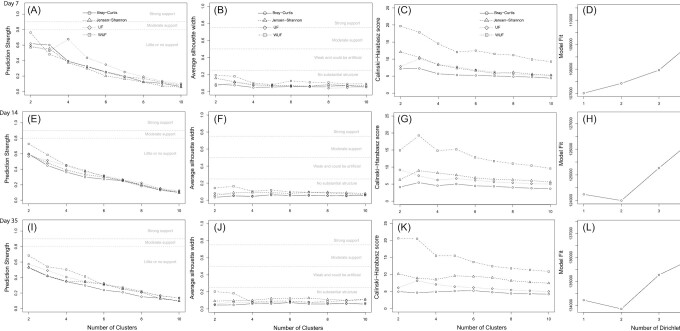
Quality and confidence scores of cluster analyses stratified by sampling day using different metrics. **(A)** In the cecal content of 7-day-old broilers, the PAM–WUF method showed near-moderate support (0.78, threshold at 0.80) for two clusters, however, the other methods showed little or no support for significance based on the prediction strength. Although there was no support for clusters, the prediction strength based on the PAM–UF method was higher for four clusters instead of two clusters. **(B)** and **(C)** Based on the silhouette index and the Calinski–Harabasz score, the PAM–UF distance metrics resulted in three clusters, and all other distance metrics in two clusters. **(D)** Based on DMM, no clusters were observed. This indicated that depending on the clustering method, the number of clusters varied in 7-day-old broilers. **(E)** On day 14, little to no support was observed for any of the distance metrics based on the prediction strength, however, the highest support was for two clusters independent of distance metrics. **(F)** No support for clusters was observed based on the silhouette index as well. **(G)** PAM–BC, –JS, and –WUF showed the highest Calinski–Harabasz score for three clusters, and PAM–UF the highest Calinski–Harabasz score for two clusters. **(H)** The DMM method resulted in two clusters. **(I)** and **(J)** In the broilers of 35 days old, also little to no support was observed for any of the distance metrics, although again the highest support was for two clusters (prediction strength, I). **(K)** The Calinski–Harabasz score showed that distance metrics BC and JS resulted in two clusters, and distance metrics UF and WUF resulted in three clusters. **(L)** The DMM method resulted in two clusters.

Commercial broilers have a short life span between hatch and slaughter of approximately 5–8 weeks, and during this period their intestinal microbiota composition changes rapidly. Therefore, the cluster analyses were also stratified by age, to identify potential clusters within age group. These analyses showed that different clustering algorithms resulted in differences in optimal cluster structures, which suggests there were no robust age-specified clusters within 7-, 14-, or 35-day-old broilers with the number of samples included in this study (Fig. 3).

### Diversity and developmental trajectories of cecal microbiota

**Figure 4.**
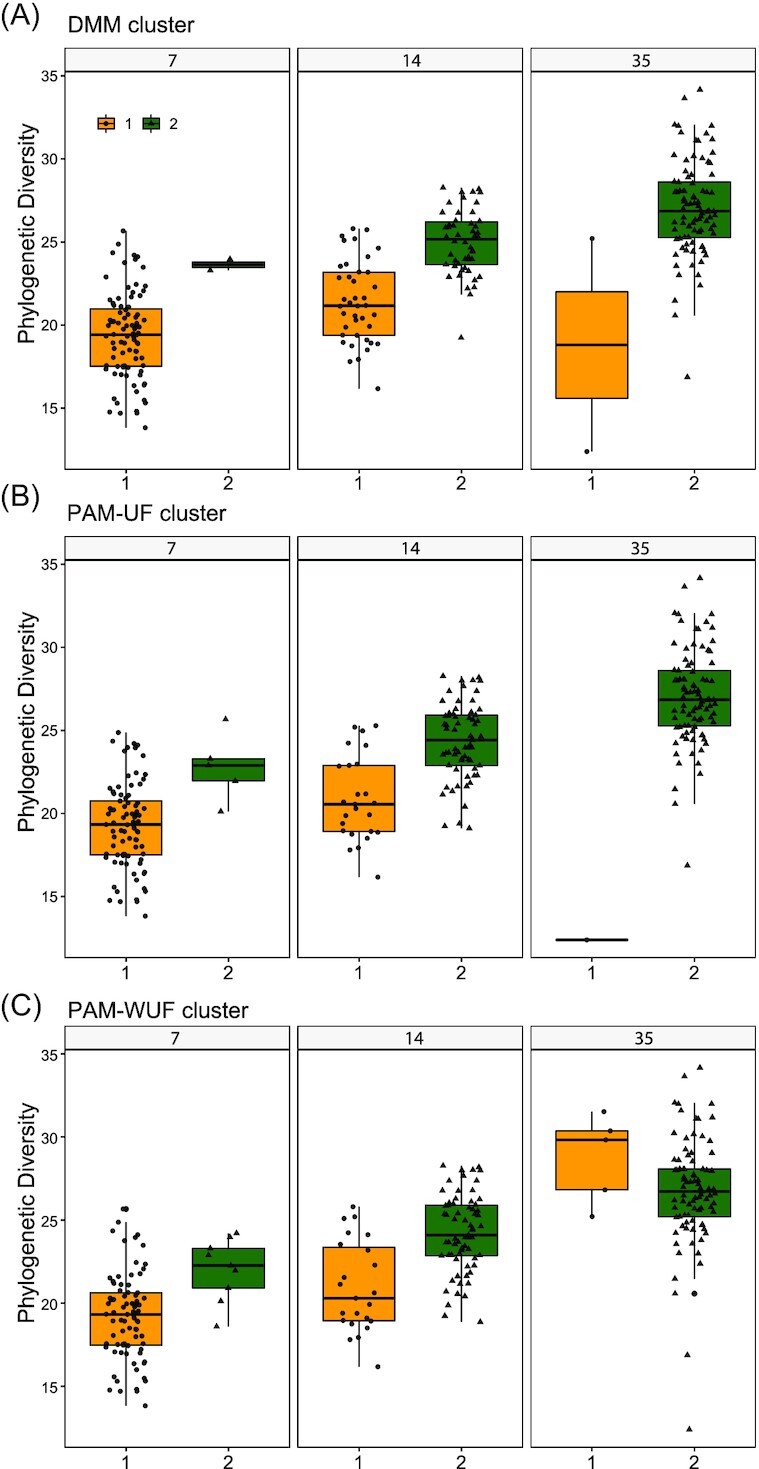
Cecal microbial alpha diversity of the two clusters stratified by age. Phylogenetic diversity (ASV level) of cecal microbiota (*n* = 270) of the two clusters using different clustering methods. **(A)** DMM cluster (Kruskal–Wallis χ2 = 161.49, *P*-value < 2.2e-16). **(B)** PAM–UF cluster,(χ2 = 151.73, *P* < 2.2e-16). **(C)** PAM–WUF cluster (χ2 = 117.35, *P* < 2.2e-16). Within broilers of 14 days old (DMM, χ2 = 36.60, *P*-value < 1.5e-09; PAM–UF, χ2 = 25.04, *P*-value < 5.6e-07; and PAM–WUF, χ2 = 19.84 *P*-value < 8.4e-06). Whiskers show 95% interval, box 50% interval.

To assess whether the two identified community types differed in composition and alpha diversity, DMM-, PAM–UF- and PAM–WUF-derived clusters were compared. Within sample (alpha) diversity defined as phylogenetic diversity was higher in cluster 2 compared to cluster 1, independent of clustering method (Fig. 4). Alternative alpha diversity metrics (ASV richness and Shannon diversity) confirmed these results (Table S2, Supporting Information). As the 14-day-old broilers were distributed across both clusters we also tested whether, within this age category, a difference between clusters could be identified. The phylogenetic diversity of 14-day-old broilers in DMM, PAM–UF-, or PAM–WUF cluster 1 was indeed lower compared to that of 14-day-old broilers in DMM cluster 2 (Fig. 4).

**Figure 5.**
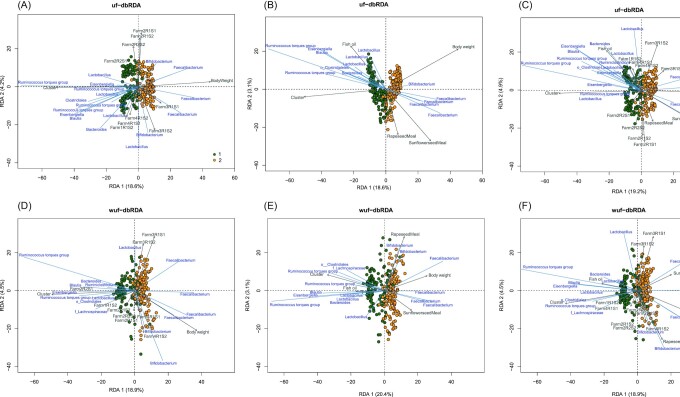
Multivariate effects of microbiota covariates on the cecal microbiota of broilers. Distance based redundancy analysis (db-RDA) triplots of the cecal microbiota of broilers with weighted and UF. Samples are colored by DMM-based cluster. **(A)** UF-db-RDA with explanatory variables cluster, flock, and body weight. **(B)** UF-db-RDA with cluster, body weight, sunflower seed meal %, rapeseed meal %, and fish oil %. **(C)** UF-db-RDA with cluster, flock, body weight, sunflower seed meal %, rapeseed meal %, and fish oil %. **(D)** WUF-db-RDA with cluster, flock, and body weight. **(E)** WUF-db-RDA with cluster, body weight, sunflower seed meal %, rapeseed meal %, and fish oil %. **(F)** WUF-db-RDA with cluster, flock, body weight, sunflower seed meal %, rapeseed meal %, and fish oil %.

We used PERMANOVA, based on UF and WUF distances, to assess the percentage of total microbiota variation the community types accounted for. This was 16.4% and 18.1% for DMM clustering, 16.5% and 17.6% for UF–PAM clustering, and 14.1% and 22.1% for WUF–PAM clustering. In addition to the two community types, 13 host and environmental characteristics that might affect composition were tested for their effect on microbiota composition using UF and WUF db-RDA (Table S3, Supporting Information). These microbiota covariates included flock size, surface (poultry house in m^2^), bird density per m^2^, litter type, age of the parent stock, hatchery, feed producer, antibiotics use, farm, and flock, as well as characteristics at the individual animal level, including body weight, age, and sex (Table S3, Supporting Information). The db-RDA analysis allowed us to determine the relative effect of these variables on microbiota composition during the development of cecal microbiota. Model 1 is was the most parsimonious UF-db-RDA model (after testing and adjusting for collinearity), and consisted of three significant explanatory variables: flock, the DMM-based community types, and body weight (Table 1). This model explained 31.7% of the cecal microbiota variation, which was very similar to the analysis based on WUF (30.6%) with the same variables (Fig. 5A and D). Only minor differences were observed with the PAM based clusters (33.3% and 31.0%). It should be noted that the two community types were related to age, and that age and body weight are highly correlated in these fast growing chickens. Variation partitioning visualized with Venn diagrams, shows this strong collinearity between cluster, body weight, and age (Figure S3, Supporting Information; 11% and 9%). Because of the collinearity between these variables, we cannot unequivocally conclude that the relative abundance of members of the genus *Faecalibacterium* was only associated with higher body weight (Fig. 5A and D), and whether the relative abundance of a member of the genus *Ruminococcus torques* group was strongly associated with the ordination in broilers within cluster, as both were also associated with the age of the broiler (Fig. 5A and D).

**Table 1.** The output of the different models based on UF db-RDA. The results are the final models. VIF scores are given for variables excluded from the full model.

**Model 1**	Df	AIC	F	Pr (> F)
Flock	9	903.10	4.51	0.005
Community types	1	893.32	13.40	0.005
Body weight	1	889.68	9.76	0.005
Surface	1	888.40	1.20	0.215
Flock size	1	882.70	0.91	0.560
Sex	1	883.65	0.96	0.580
Feed producer	2	881.65		
Litter type	2	881.65		
VIF scores: age parent stock (1191), age (164), density (22), farm (11), hatchery (11), and antibiotic use (6).				
**Model 2**	**Df**	**AIC**	**F**	**Pr (> F)**
Community types	1	898.65	13.26	0.005
Body weight	1	894.62	9.16	0.005
Rapeseed meal %	1	894.18	8.71	0.005
Sunflower seed meal %	1	893.14	7.67	0.005
Fish oil %	1	890.60	5.12	0.005
VIF scores: soybean meal % (1580), farmers wheat % (309), metabolizable energy(AME)·kg^−1^ (153), fecal digestible lysine g·kg^−1^ (13 449), maize % (195), oats % (2998), phosphorous (22), wheat % (42), potato protein % (9), methionine + cysteine (5), and flock (4)				
**Model 3**	**Df**	**AIC**	**F**	**Pr (> F)**
Flock	9	887.41	4.13	0.005
Body weight	1	877.32	10.26	0.005
Community types	1	876.49	9.44	0.005
Rapeseed meal %	1	873.11	6.16	0.005
Sunflower seed meal %	1	873.07	6.11	0.005
Fish oil %	1	870.75	3.89	0.005
VIF scores: soybean meal % (1580), farmers wheat % (309), metabolizable energy (AME)·kg^−1^ (153), fecal digestible lysine g·kg^−1^ (13 449), maize % (195), oats % (2998), phosphorous (22), wheat % (42), potato protein % (9), and methionine + cysteine (5)				

To assess the impact of differences in feed components on the cecal microbiota, 13 feed components were added as explanatory variables (Table S3, Supporting Information). In model 2, only body weight, sunflower seed meal %, rapeseed meal %, and fish oil % were related to microbiota composition and together with the DMM community types explained 26.4% (UF-db-RDA) and 27.9% (WUF-db-RDA) of cecal microbiota variation (Table 1, Fig. 5B and E). Model 2 did not contain flock anymore, however, when flock was included in the model (model 3), it did increase the explanatory power to 37.0% and 36.7% (UF and WUF; Fig. 5C and F). The addition of flock resulted in a variance inflation factor (VIF) value of 4.2, suggesting that the variable flock contains limited or no unique information. In this dataset, it is difficult to disentangle the contribution of the different feed components from flock and farm effects, because dietary components were strongly correlated with farms. For instance, Fig. 5(C) and (D), show that e.g. fish oil % was associated with Farm 1 and rapeseed meal % with Farm 2.

**Figure 6.**
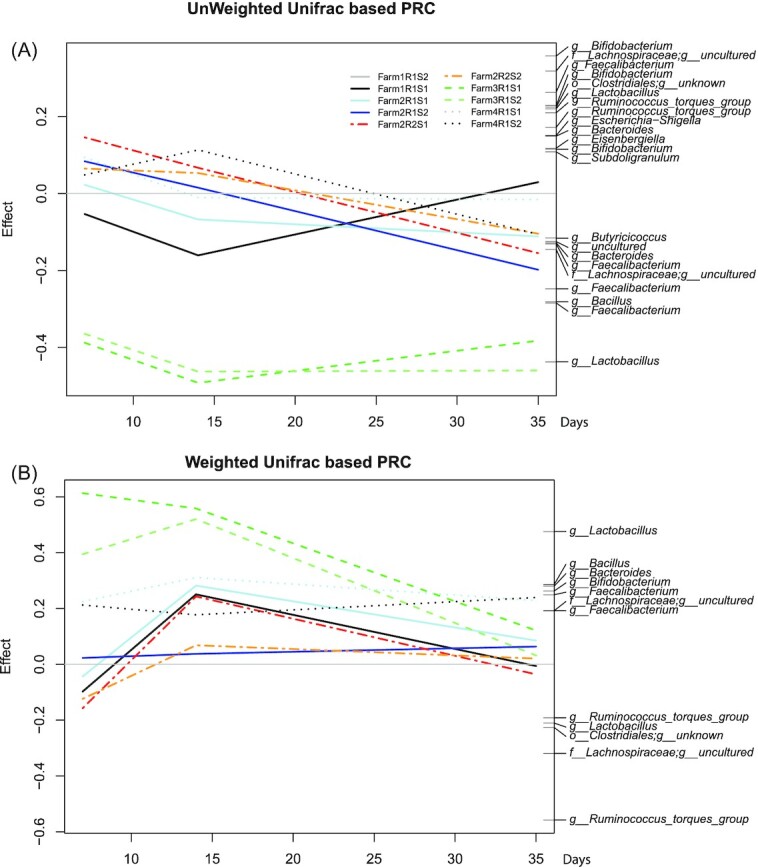
Multivariate temporal compositional dynamics of cecal microbiota in different farms and houses. PRC analysis (*n* = 270) based on unweighted **(A)** and weighted **(B)** UniFrac distances summarizing the multivariate comparison of cecal broiler microbiota in different farms and houses over time. Each line corresponds to the cecal microbiota of a different poultry flock. The gray straight line at zero represents the temporal microbiota dynamics of Farm 1, poultry flock 2, and serves as the reference. ASVs with large differences compared to the reference have large effect sizes while ASVs with equal presence have zero weight. Taxa plotted vertically on the right axis are the main drivers of the differences between flocks. Those with negative weights follow the negative lines, whereas those with positive weights follow the opposite pattern.

Finally, to determine the development of cecal microbiota composition of poultry flocks in different farms over time was visualized using Principle Response Curve (PRC; Fig. 6). When only considering community membership using UF, the cecal microbiota at all farms was relatively similar on day 7, except for Farm 3 (Fig. 6A), which remained distinct, due to the presence of certain *Lactobacillus* ASV (Fig. 6A). The WUF based PRC, which also considers the abundance of ASV, showed that Farms 3 and 4 started with a deviant composition from Farms 1 and 2. Through time the composition of Farm 3 converged, but the composition of Farm 4 did not (Fig. 6B). Taken together, we observed that feed components, flock, and body weight had an effect on the cecal microbiota composition. Importantly, we observed a convergence of cecal broiler microbiota through time, showing a conserved developmental pattern exemplified by the identified clusters. However, especially feed associated variables were associated with the farm, therefore, the independent effects of these variables on microbiota composition could not be disentangled further.

## Discussion

This study showed that cecal microbiota of broilers from commercial broiler farms could be stratified into community types and that cecal microbiota development patterns are conserved between farms and flock across age, despite the use of different farms and flocks. We identified, two robust community types; one dominated by broilers of 7 days old, and one dominated by broilers of 35 days old. Broilers, 14-day-old, were divided across both community types. This indicates that the development of the cecal microbiota followed a general trajectory across farms, and that a transition occurred around the second week of life.

The development of the intestinal microbiota toward a matured intestinal microbiota in chickens is not fully understood (Yin et al. 2010, Ballou et al. 2016, Donaldson et al. 2017, Jurburg et al. 2019, Kers et al. 2019). Early life colonization, feed additives, and antimicrobial drugs play a large role in microbiota composition and affect the development (Ballou et al. 2016, Gao et al. 2017). Gao et al., 2017 showed maturation of the fecal microbiota until day 30. Our results indicate that the temporal cecal microbiota development of broiler chickens undergoes two distinct phases, independent of factors as farm or flock, while in human infants, 10 clusters, and three phases have been observed (Stewart et al. 2018). The observation that the broilers of 14 days old were divided across both clusters suggests that the period around the second week of life is an important transitional phase. In contrast to other studies that suggest stabilization of the community richness (alpha diversity) by day 14 in cecal or fecal content (Lu et al. 2003, Jurburg et al. 2019), our data showed that community richness was still increasing around this age. This may suggest that in the other experimental studies this transition phase was earlier, compared to our field study. However, considering the large time interval between days 14 and 35 the exact moment of stabilization could not be determined in this study and may have occurred shortly after day 14.

In general, identifying community types is sensitive to the applied methodologies (Koren et al. 2013). The highest prediction strength, silhouette index, and Calinski–Harabasz score were observed with the PAM–WUF method. This is in line with previous suggestions that WUF distance metric might be the best choice for cluster or enterotyping structures (Koren et al. 2013). Our results showed high to moderate support for two community types within the total dataset based on the prediction strength, but the silhouette index did not support this observation. This trend of high to moderate support based on the prediction strength and a limited support based on the silhouette index has been observed before. It has been noticed that prediction strength is most sensitive to observe community types (Koren et al. 2013, Yuan et al. 2020). In previous studies, four fecal community types and three duodenum community types were identified in broiler chickens of 57 days (*n* = 31) and 77 days old (*n* = 206) (Kaakoush et al. 2014, Yuan et al. 2020). In contrast, we observed no robust community types in age-stratified analyses of 7-, 14-, or 35-day-old broilers. This might be because in our study cecal content was used, and the broilers were much younger. In humans, the establishment of intestinal microbiota cluster structures has been estimated to occur between the age of 9 and 36 months (Bergström et al. 2014, Zhong et al. 2019). Furthermore, the limited number of individual samples in the stratified data analysis (*n* = 90) might also be an explanation why we did not observe robust community types within age groups.

Previous research in broilers showed that continuous supply of in-feed antibiotics decreased the maturation of the intestinal microbiota, while feed with the probiotic bacterium *Lactobacillus plantarum* accelerated the development of intestinal microbiota (Gao et al. 2017). On days 3 until 6 of the production cycle of Farm 3, the flock in poultry house 2 received a 3-day antibiotic treatment (trimethoprim + sulfamethoxazol). This early life antibiotic treatment did not result in different community types. Although the PRC analysis showed that Farm 3 (poultry houses 1 and 2), was distinct from the other farms on day 7, this was due to the higher relative abundance of a member of the genus *Lactobacillus* (ASV). This higher relative abundance of *Lactobacillus* was also observed on days 14 and 35 in house 2 as well in house 1, where no treatment with antibiotics was applied. This suggests that the difference between Farm 3 and the other farms could not be solely attributed to the early life treatment with antibiotics, or that the houses were not distinct due to transmission of microbes between poultry houses. Alternatively, it can also be argued that this deviance from other farms was more related to lower chick health on Farm 3, and not to antibiotic treatment. We cannot rule out that the effects on microbiota were mostly caused by a lower chick quality and disease in both houses than by the use of antibiotics. In both houses on the farm, the day old chicks were obtained from the same hatchery and parent flock and both had relatively high early life mortality due to omphalitis. In house 2, slightly higher mortality was observed compared to house 2, which prompted the antibiotic use in this house alone.

In addition to the observed community types, body weight, and the percentage of the feed components sunflower seed meal, rapeseed meal, and fish oil explained 26%–27% of the cecal microbiota variation between broilers. However, when flock was included in the analysis, the explained variation increased by around 10% but so did the collinearity between variables. This indicates that it is difficult to disentangle the contribution of the different feed components from the effect of flock and farm, unless a larger number of farms, with similar feeds would be included in the analysis. Another study identified four fecal community types in two farms and suggested the grouping did not occur by chance because considerable microbiota variation between farms was observed (Kaakoush et al. 2014). In our study, the two community types were observed across different poultry flocks. All flocks were located in the Netherlands, where similar feed compounds are available from different feed suppliers, and all farms used wheat-based feeds. Therefore, the variation between feed and flock is relatively small, which may limit the generalizability of the relevance of the identified feed compounds to other countries.

In humans, diet can provoke a shift in intestinal microbiota clusters (Wu et al. 2011, Kovatcheva-Datchary et al. 2015). Although the feed in different farms was obtained from different feed suppliers, this was not reflected in our developmental trajectory. During the production cycle, feed shifts occurred on days 9 (Farms 1 and 2), 10 (Farms 2 and 4), and 12 (Farm 3), however, with a retention time of the digestive tract of less than 12 h (McWhorter et al. 2009, Sundu 2009), we assume that the microbiota was already adjusted to the feed shift before day 14. Also, if feed or farm would be the main drivers of the temporal development, then the optimal number of community types (clusters) would have been three, associated with broiler age, or four associated with the farm.

Only a few studies have focused on providing insight into the factors that influence the temporal development and microbiota configurations of broiler chicken intestinal microbiota (Johnson et al. 2018). This research remains challenging, because of the wide array of available approaches, each with their advantages and disadvantages (Costea et al. 2018). No single method is perfect, and although the db-RDA resulted in the same important factors, a less stringent VIF threshold influences the results and increases explained variation (Zuur et al. 2010).

In summary, stratification of microbiota composition in clusters showed two robust community types in the cecal microbiota of broiler chickens and with the PRC results, this indicates a conserved developmental trajectory of the cecal microbiota across 10 different commercial broiler flocks on four different farms. This emphasizes the importance for further investigation of mechanisms underlying microbiota development and functions that affect broiler health and performance. This mechanistic knowledge can contribute to the development of new nutritional interventions, improved management, as well as better diagnostic tools to improve broiler health.

## Materials and methods

### Ethical statement

The field study was approved by the Dutch Central Authority for Scientific Procedures on Animals and the Animal Experiments Committee (registration number AVD108002016442) and was carried out in compliance with all relevant legislation.

### Farm selection and data collection

Data for this study were obtained from four broiler farms in the Netherlands, each with two similar houses. All farms had conventional Ross 308 broilers, both male and female. The farms were selected for good production performance, as we were interested in a healthy intestinal microbiota. Also, to reduce the chance of including flocks treated with antibiotics, only farms with an antimicrobial use in the previous months below 15 DDDAf (defined daily dose per animal year on farm level) were recruited for the study. All selected farms were within the target zone for antimicrobial use according to national benchmark thresholds for poultry farms in 2017(SDa 2018). Notwithstanding, two flocks were treated with antibiotics. The flock in poultry house 2 of Farm 3 received a 3-day antibiotic treatment (trimethoprim + sulfamethoxazol) between days 3 and 6 of the production cycle, and the flock in poultry house 2 of Farm 4 received a 3-day antibiotic treatment (amoxicillin) from day 22 onward of the production cycle (Table 2). The farms were visited on days 7, 14, and 35 of the production cycle. On one of the farms (Farm 2), data was collected from two consecutive production cycles. The farms received chicks from different commercial hatcheries. An overview of the different farm characteristics of the poultry flocks is provided in Table 2. The diets on all farms were provided *ad libitum* and were mostly wheat-based, but there were differences in composition of the feed and feed suppliers between farms. Table 2 provides an overview of the feed details per time point. Coccidiostatic drugs were standardly applied in all flocks (Table 2).

**Table 2.** Farm characteristics.

	Farm 1		Farm 2 cycle 1		Farm 2 cycle 2		Farm 3		Farm 4	
Start production cycle (visit)	August 2016		June 2017		June 2017		August 2017		August 2017	
Hatchery	Hatchery A		Hatchery B		Hatchery B		Hatchery C		Hatchery D	
Age of the parent flock (weeks)	55		35		42		49		54	
Type of litter	Wood shavings		Peat		Peat		Straw pellets		Straw pellets	
Feed supplier	Supplier A		Supplier B		Supplier B		Supplier C		Supplier B	
Poultry house	1	2	1	2	1	2	1	2	1	2
Size poultry house (m^2^)	1313	1313	972	968	972	968	1600	1850	1350	1730
Flock size	28 000	28 000	23 500	23 500	23 500	23 500	32 200	38 400	30 500	39 000
Antibiotic treatment	No	No	No	No	No	No	No	Yes (day 3)	No	Yes (day 22)

The broilers of Farm 2 cycle 1 and 2 are of the same parent flock. Each poultry house contains one broiler flock.

### Cecal content collection and 16S rRNA gene amplicon sequencing

At each visit, nine broilers were randomly selected for sampling from each poultry house. Coveralls, footwear, and all sampling materials were changed between sampling of the two poultry flocks on the same farm. The start of the sampling of broilers took place at least 30 min after the end of a dark-period, to avoid low amounts of content in the intestinal tract at sampling. Broilers were individually weighed, checked for abnormalities, and euthanized by cervical dislocation. The gastrointestinal tract was quickly but carefully removed, using a procedure that was as sterile as possible, as previously described in detail (Kers et al. 2019). The cecal content was stored at −80°C before extraction of DNA as previously described (Kers et al. 2019). Briefly, DNA was extracted from 0.25 g cecal content, using 700 μl of Stool Transport and Recovery (STAR) buffer (Roche Diagnostics Nederland BV, the Netherlands) and repeated bead beating. The PCR reactions contained 36.5 μl nucleotide free water (Promega, USA), 0.5 μl of 2 U μl^−1^ polymerase, 10 μl of 5 × HF buffer, 1 μl of 10 μM stock solutions of each of the forward and reverse primers, 1 μl 10 mM dNTPs (Promega), and 1 μl template DNA. Reactions were held at 98°C for 30 s and amplification proceeded for 25 cycles at 98°C for 10 s, 42°C for 10 s, 72°C for 10 s, and a final extension of 7 min at 72°C. DNA concentration was measured with a NanoDrop ND-1000 spectrophotometer (NanoDrop® Technologies, USA), and samples were stored at −20°C until further use. Barcoded amplicons covering the variable regions V5–V6, were generated by PCR amplification of the bacterial 16S rRNA gene using barcoded primers 784F and 1064R as described before (Ramiro-Garcia et al. 2016). To ensure high quality sequencing data, synthetic communities of known composition were used as positive controls (Ramiro-Garcia et al. 2016), and nuclease free water as negative controls. Sequencing of resulting libraries was performed by GATC GmbH (now part of Eurofins Genomics Germany GmbH, Konstanz, Germany) on an Illumina Hiseq2500 instrument. Raw sequence data was analyzed using NG-tax 2.0 (Poncheewin et al. 2020) using SILVA 128 as 16S rRNA gene reference database to assign taxonomy (Quast et al. 2013).

### Statistical analysis

Statistical analyses were performed in R, version 3.4.3 (R Core Team 2017), using packages: Phyloseq, Microbiome, Vegan, DirichletMultinomial, and RVAideMemoire (Oksanen 2010, McMurdie and Holmes 2013, Morgan 2014, Lahti 2017, Pinheiro et al. 2018, Hervé and Hervé 2020).

Clustering was performed according to PAM-based protocols using JSD (PAM–JSD) at ASV-level (Arumugam et al. 2011), BC (PAM–BC), UF (PAM–UF), and WUF (PAM–WUF). The optimal number of clusters was calculated using Prediction Strength, Average Silhouette Width (silhouette index), Calinski–Harabasz index, and Laplace approximation (Holmes et al. 2012, Koren et al. 2013). Also, DMM, a probabilistic model, was applied to cluster the 16S rRNA gene sequence data at ASV-level (Holmes et al. 2012). To test for differences in relative abundance of genera between clusters, Wilcoxon rank-sum test corrected for multiple comparisons using Benjamini–Hochberg (BH) was used. A corrected *P-*value (q-value) of < .05 was considered statistically significant.

Shannon diversity, ASV richness and Faith’s phylogenetic diversity (Faith 2006) were calculated to define microbial alpha diversity. Differences in alpha diversity were tested with a Kruskal–Wallis test, and pairwise comparisons were tested using Wilcoxon rank-sum tests and corrected for multiple testing with BH. Beta diversity was visualized using principal coordinates analysis (PCoA), and nonparametric permutational analysis of variance (PERMANOVA) was used to analyze multivariate impact of the clusters on the microbiota (Anderson 2001) a *P*-value of < .05 was considered significant.

To examine the multivariate differences between microbiota compositions of poultry houses over time, distance based principal response curve (PRC) analysis was used (Shankar et al. 2017), PRC was originally developed to analyze time-series data and carries out partial redundancy analysis (RDA) ordination to obtain estimates of community changes using time as a predictor variable (Van den Brink and Braak 1999).

In addition, to determine which explanatory variables impact broiler cecal microbiota, db-RDA, based on WUF and UF distances were performed on ASV level data. To determine the most parsimonious model, the model that explained the microbiota variation with the least number of explanatory variables, a stepwise selection (both directions) was used based on the Akaike information criterion (AIC), using 9999 number of iteration steps of dropping and adding terms. The most parsimonious model were tested for collinearity and those with a VIF > 3 were removed from the model one by one (Zuur et al. 2010). High VIF values suggest that the variable contains limited or no unique information, and therefore, is redundant in the set of explanatory variables.

## Data availability

Sequence data was deposited into the Sequence Read Archive (SRA) at the NCBI under accession number PRJNA844268. The data files and R-scripts can be accessed through the Github page: https://github.com/mibwurrepo/Kers_etal_ConservedDevelopment.

## Authors’ Contributions

Conceptualization: F.C.V., J.A.S., and H.S.; methodology: F.C.V., J.A.S., E.A.J.F., J.G.K., and H.S.; investigation: J.G.K. and F.C.V.; data analysis: J.G.K and G.D.A.H; writing original draft: J.G.K.; writing, review, and editing: G.D.A.H, F.C.V., J.A.S., E.A.J.F., and H.S.; funding acquisition: F.C.V.; and supervision: F.C.V., J.A.S., E.A.J.F., and H.S.

## Supplementary Material

fiac090_Supplemental_File
