# Supplementary material for: Conserved developmental trajectories of the cecal microbiota of broiler chickens in a field study
Source: FEMS Microbiol Ecol. 2022 Jul 25;98(9):fiac090. doi: 10.1093/femsec/fiac090 (PMC9423033; doi:10.1093/femsec/fiac090)
Supplement: fiac090_Supplemental_File [file fiac090_supplemental_file.docx]

# **Supplemental material**

**Figure S1:** Cumulative relative abundance (%) of top 21 microbial genera for two clusters based on the five different cluster methods, A= cluster one, B = cluster two. In total 270 broilers of 7, 14 or 35 days old, BC = Bray Curtis distance, JS = Jensen Shannon distance, UF = unweighted UniFrac distance, WUF = Weighted UniFrac distance, and DMM = Dirichlet Multinomial Mixtures


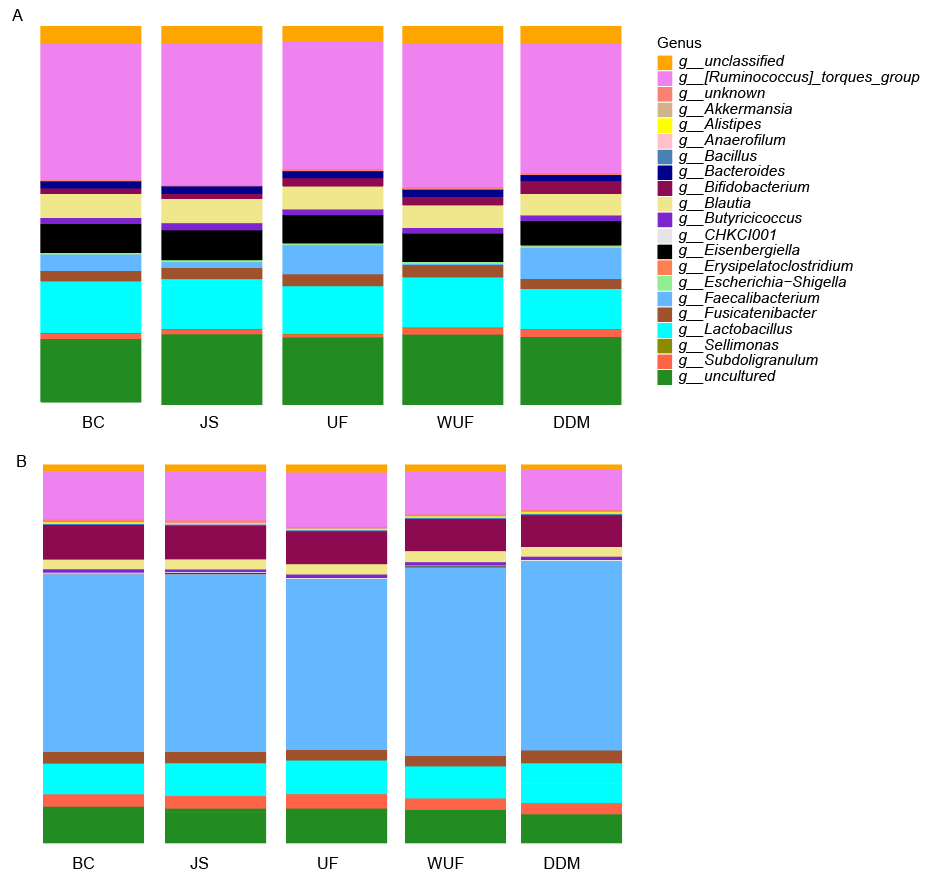


**Figure S2:** Principal coordinate analysis of cecal broiler microbiota A) unweighted UniFrac and B) weighted UniFrac. Different colors indicate different sampling days, and different shapes indicated different farms.


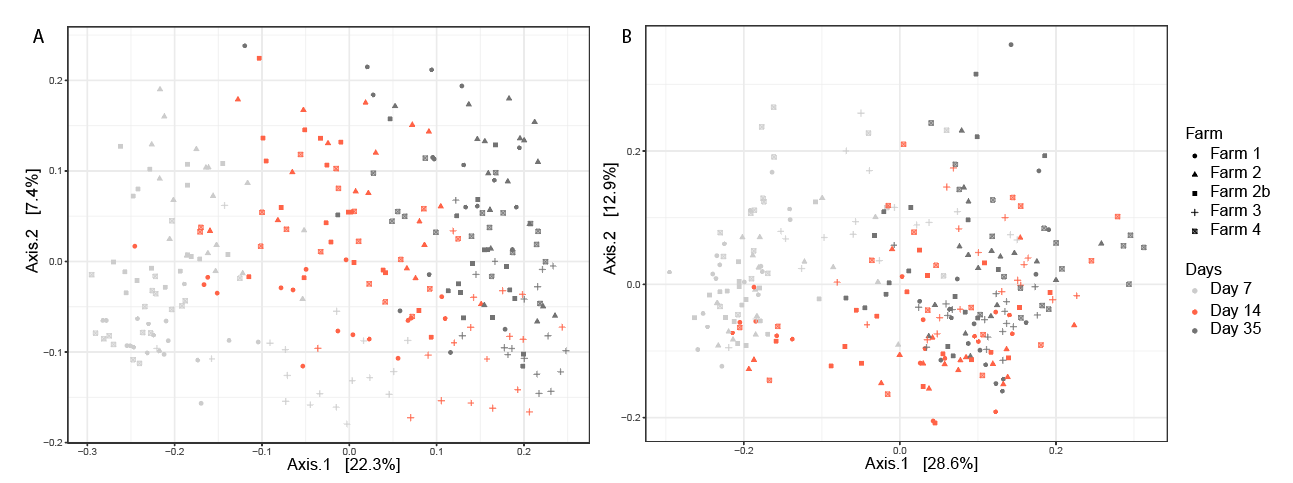


**Figure S3:** Variation partitioning based Venn diagrams**.** Venn diagrams displaying the partitioning of the variation over different microbiota covariates uf-db-RDA (A) and wuf-db-RDA (B).

##


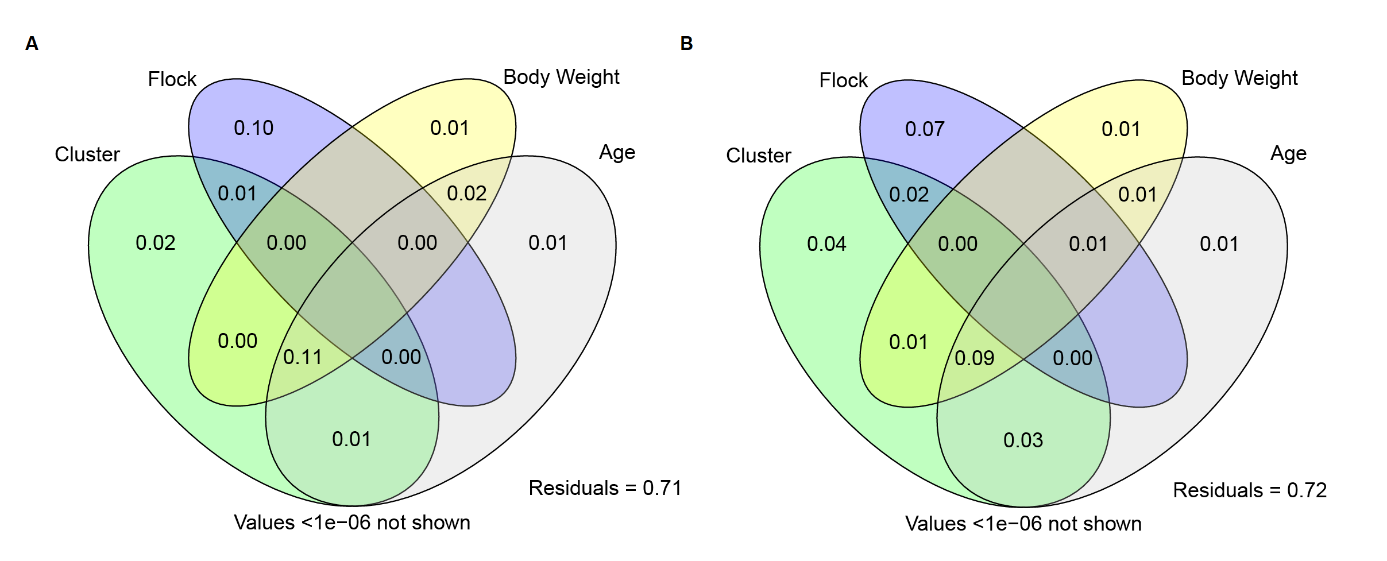


**Table S1:** Top 25 ASVs whose relative abundance differed significantly between two clusters identified by different clustering methods. Significantly different ASVs between cluster 1 and cluster 2, across different methods (unpaired Wilcoxon rank-sum test. q <0.05). Bold names indicate taxa that were ambiguous between methods. (+) indicates an increase and (-) a decrease in relative abundance of cluster 1 compared to cluster 2.

| Top | DMM | | **%** | | PAR-UF | | PAM-WUF | | PAM-BC | | PAM-JS | |
| --- | --- | --- | --- | --- | --- | --- | --- | --- | --- | --- | --- | --- |
| 1 | g_Faecalibacterium | **+** | | g_Faecalibacterium | | g_Faecalibacterium | | g_Faecalibacterium | | g_Faecalibacterium | |  |
| 2 | g_Ruminococcus torques | **-** | | g_Ruminococcus torques | | g_Ruminococcus torques | | g_Ruminococcus torques | | g_Ruminococcus torques | |  |
| 3 | g_Eisenbergiella | **-** | | g_Eisenbergiella | | g_Eisenbergiella | | g_Eisenbergiella | | g_Eisenbergiella | |  |
| 4 | o_Clostridiales | **-** | | o_Clostridiales | | o_Clostridiales | | g_Bifidobacterium | | o_Clostridiales | |  |
| 5 | f_Lachnospiraceae | **-** | | f_Lachnospiraceae | | f_Lachnospiraceae | | o_Clostridiales | | f_Lachnospiraceae | |  |
| 6 | f_Lachnospiraceae | **-** | | g_Bifidobacterium | | g_Bifidobacterium | | f_Lachnospiraceae | | g_Bifidobacterium | |  |
| 7 | g_Bifidobacterium | **+** | | g_Lactobacillus | | g_Lactobacillus | | g_Lactobacillus | | g_Lactobacillus | |  |
| 8 | g_Bacteroides | **-** | | g_Subdoligranulum | | f_Lachnospiraceae | | g_Bacteroides | | f_Lachnospiraceae | |  |
| 9 | g_Subdoligranulum | **+** | | g_Bacteroides | | g_Bacteroides | | f_Lachnospiraceae | | g_Bacteroides | |  |
| 10 | g_GCA-900066575 | **-** | | f_Lachnospiraceae | | g_Subdoligranulum | | g_Subdoligranulum | | g_Subdoligranulum | |  |
| 11 | g_Alistipes | **+** | | g_Alistipes | | g_GCA-900066575 | | **g_Blautia** | | g_CA-900066575 | |  |
| 12 | g_Escherichia-Shigella | **-** | | g_ GCA-900066575 | | g_Alistipes | | g_GCA-900066575 | | g_Alistipes | |  |
| 13 | g_Fusicatenibacter | **+** | | g_Escherichia-Shigella | | g_Lachnoclostridium | | g_Alistipes | | g_Lachnoclostridium | |  |
| 14 | o_Clostridiales | **-** | | g_Lachnoclostridium | | o_Clostridiales | | g_Lachnoclostridium | | g_Escherichia-Shigella | |  |
| 15 | g_Lachnoclostridium | **-** | | o_Clostridiales | | g_Escherichia-Shigella | | g_Escherichia-Shigella | | o_Clostridiales | |  |
| 16 | g_Erysipelatoclostridium | **-** | | g_Sellimonas | | g_Sellimonas | | o_Clostridiales | | g_Erysipelatoclostridium | |  |
| 17 | g_Christensenellaceae | **+** | | g_Ruminococcaceae_UCG-14 | | g_Bacillus | | g_Sellimonas | | g_Sellimonas | |  |
| 18 | g_Bacillus | **+** | | g_Ruminococcaceae_UCG-005 | | g_Erysipelatoclostridium | | g_Fusicatenibacter | | g_Fusicatenibacter | |  |
| 19 | g_Ruminococcaceae_UCG-14 | **+** | | g_Erysipelatoclostridium | | g_Ruminococcaceae_UCG-14 | | g_Bacillus | | g_Ruminococcaceae_UCG-14 | |  |
| 20 | g_Ruminococcaceae_UCG-005 | **+** | | g_Fusicatenibacter | | g_Ruminococcaceae_UCG-005 | | g_Ruminococcaceae_UCG-14 | | g_Ruminococcaceae_UCG-005 | |  |
| 21 | g_Sellimonas | **-** | | g_Christensenellaceae | | g_Christensenellaceae | | g_Ruminococcaceae_UCG-005 | | g_Christensenellaceae | |  |
| 22 | g_**Ruminiclostridium** | **-** | | g_Bacillus | | g_**Ruminiclostridium** | | g_Erysipelatoclostridium | | g_Bacillus | |  |
| 23 | g_**Akkermansia** | **+** | | g_uncultured_bacterium | | g_Fusicatenibacter | | g_Christensenellaceae | | g_Butyricicoccus | |  |
| 24 | g_**Ruminiclostridium** | **+** | | g_**Ruminiclostridium** | | g_**Akkermansia** | | g_uncultured_bacterium | | g_uncultured_bacterium | |  |
| 25 | g_uncultured_bacterium | **+** | | g_**Akkermansia** | | g_uncultured_bacterium | | g_**Ruminiclostridium** | | g_**Akkermansia** | |  |

## Table S2: Differences in alpha diversity were tested with a Kruskal-Wallis test.

| **Community type 1 vs 2** |  | |  | |
| --- | --- | --- | --- | --- |
| ASV richness | χ2 | | *p – value* | |
| DMM cluster | 109.67 | | < 2.2e-16 | |
| PAM-UF | 88.06 | | < 2.2e-16 | |
| PAM-WUF | 74.33 | | < 2.2e-16 | |
| Age | 85.86 | | < 2.2e-16 | |
| Shannon diversity | χ2 | | *p = value* | |
| DMM cluster | 47.19 | | 6.5e-12 | |
| PAM-UF | 39.85 | | 2.7e-10 | |
| PAM-WUF | 32.82 | | 1.0e-08 | |
| Age | 36.81 | | 1.0e-08 | |
|  |  | |  | |
| **Age 7 vs 14 & 14 & 35** | Day 7 vs 14 | | Day 14 vs 35 | |
| Phylogenetic diversity | χ2 *p – value* | | χ2  *p – value* | |
| DMM cluster 1 | **14.43** | **1.5e-4** | 0.03 | 0.859 |
| DMM cluster 2 | 1.10 | 0.295 | **20.65** | **5.5e-06** |
| PAM-UF cluster 1 | **6.01** | **0.014** | 2.78 | 0.096 |
| PAM-UF cluster 2 | 2.44 | 0.118 | **33.64** | **6.6e-09** |
| PAM-WUF cluster 1 | **5.76** | **0.016** | **11.49** | **7.0e-4** |
| PAM-WUF cluster 2 | **6.33** | **0.012** | **31.63** | **1.8e-08** |
| ASV richness |  |  |  |  |
| DMM cluster 1 | 0.96 | 0.326 | 0.03 | 0.859 |
| DMM cluster 2 | 2.47 | 0.116 | 2.66 | 0.103 |
| PAM-UF cluster 1 | 0.71 | 0.400 | 2.78 | 0.095 |
| PAM-UF cluster 2 | 3.21 | 0.073 | **11.03** | **8.9e-4** |
| PAM-WUF cluster 1 | 0.02 | 0.885 | **11.09** | **8.7e-4** |
| PAM-WUF cluster 2 | **4.89** | **0.027** | **7.63** | **5.8e-3** |
| Shannon diversity |  |  |  |  |
| DMM cluster 1 | 0.02 | 0.902 | 0.01 | 0.906 |
| DMM cluster 2 | 0.44 | 0.505 | 2.18 | 0.140 |
| PAM-UF cluster 1 | 0.03 | 0.856 | 2.78 | 0.096 |
| PAM-UF cluster 2 | 1.3e-4 | 0.991 | **6.99** | **8.2e-3** |
| PAM-WUF cluster 1 | 0.43 | 0.513 | **11.08** | **8.7e-4** |
| PAM-WUF cluster 2 | 0.13 | 0.718 | 3.25 | 0.072 |

##

## Table S3: Explanatory variables (host, environmental and feed components)

| ***Host and environmental characteristic (n=13)*** |
| --- |
| Farm (4 farms), age (7, 14, 35), body weight, flock (n=10), sex, age parent stock, hatchery, litter type, flock size, density (number of animals per m^2^), Surface (poultry house in m^2^), feed producer, antibiotic use (yes/no) |
| ***Feed Components (n=13)*** |
| Wheat %, Farmers wheat %, Maize %, Oats %, Soybean meal %, Potato protein %, Rapeseed meal %, Sunflower seed meal %, Fish oil %, Metabolizable energy (AME)·kg− 1, Phosphorous, Fecal digestible lysine g·kg− 1, Methionine + Cysteine |

## Table S4: Feed characteristics and coccidiostats

|  | Farm 1 | Farm 2  Cycle 1 | Farm 2  Cycle 2 | Farm 3 | Farm 4 |
| --- | --- | --- | --- | --- | --- |
| **Day 7** | | | | | |
| Wheat %  Farmers wheat % | 34  0 | 42  0 | 42  0 | 40  0 | 47  0 |
| Maize % | 22.2 | 20 | 20 | 25 | 17 |
| Oats % | 9.5 | 1.5 | 1.5 | 5.0 | 1.5 |
| Soybean meal % | 23.1 | 28.0 | 28.0 | 19 | 24 |
| Potato protein % | 1.0 | 0 | 0 | 0.5 | 0 |
| Rapeseed meal % | 1.5 | 0 | 0 | 0 | 0 |
| Sunflower seed meal % | 0 | 0 | 0 | 2 | 0 |
| Fish oil % | 0.4 | 0 | 0 | 0 | 0 |
| Metabolizable energy (AME)·kg− 1 | 2970 kcal | 2925 kcal | 2925 kcal | 2945 kcal | 2925 kcal |
| Phosphorous | 3.9 | 5.4 | 5.4 | 3.48 | 4.5 |
| Fecal digestible lysine g·kg− 1 | 12.6 | 11.8 | 11.8 | 10.3 | 11.4 |
| Methionine + Cysteine | 8.5 | 8.7 | 8.7 | 7.82 | 8.4 |
| Coccidiostatic drugs | Narasin and nicarbazin | Narasin and nicarbazin | Narasin and nicarbazin | Narasin and nicarbazin | Narasin and  nicarbazin |
| **Day 14** | | | | | |
| Wheat %  Farmers wheat % | 43 12 | 55 11 | 55 11 | 43  16 | 42  10 \| 12 |
| Maize % | 20.4 | 7.5 | 7.5 | 20 | 20 |
| Oats % | 4.0 | 2.0 | 2.0 | 5.0 | 3.0 |
| Soybean meal % | 22.9 | 20.0 | 20.0 | 19 | 22.0 |
| Potato protein % | 0.3 | 0 | 0 | 0 | 0 |
| Rapeseed meal % | 1.0 | 3.0 | 3.0 | 0 | 2.0 |
| Sunflower seed meal % | 0 | 3.5 | 3.5 | 2.5 | 0 |
| Fish oil % | 0 | 0 | 0 | 0 | 0 |
| Metabolizable energy (AME)·kg− 1 | 3000 kcal | 2935 kcal | 2935 kcal | 3020 kcal | 2925 kcal |
| Phosphorous | 3.7 | 3.8 | 3.8 | 3.12 | 3.3 |
| Fecal digestible lysine g·kg− 1 | 12.1 | 11.1 | 11.1 | 10.2 | 10.4 |
| Methionine + Cysteine | 8 | 8.3 | 8.3 | 7.9 | 7.8 |
| Coccidiostatic drugs | Narasin and nicarbazin | Narasin and nicarbazin | Narasin and nicarbazin | Narasin and nicarbazin | Salinomycin |
| **Day 35** | | | | | |
| Wheat %  Farmers wheat % | 48 37 | 62 25 | 62 25 | 53  35 | 55  34 |
| Maize % | 18.5 | 8.0 | 8.0 | 15 | 10 |
| Oats % | 0 | 0 | 0 | 2.5 | 0 |
| Soybean meal % | 19.9 | 17.5 | 17.5 | 18 | 21.0 |
| Potato protein % | 0 | 0 | 0 | 0 | 0 |
| Rapeseed meal % | 4.5 | 0 | 0 | 0 | 2 |
| Sunflower seed meal % | 0 | 2.8 | 2.8 | 2.5 | 0 |
| Fish oil % | 0 | 0 | 0 | 0 | 0 |
| Metabolizable energy (AME)·kg− 1 | 3050 kcal | 3025 kcal | 3025 kcal | 3070 kcal | 3025 kcal |
| Phosphorous | 3.2 | 2.9 | 2.9 | 2.8 | 2.9 |
| Fecal digestible lysine g·kg− 1 | 10.7 | 9.4 | 9.4 | 10.0 | 10.0 |
| Methionine + Cysteine | 7 | 7.2 | 7.2 | 7.8 | 7.6 |
| Coccidiostatic drugs | Narasin | none | none | none | none |

**Legend:** Feed characteristics across age and farm.
